# Merit-Based Incentive Payment System: longitudinal performance and uneven rewards for safety-net providers over 5 years

**DOI:** 10.1093/haschl/qxaf105

**Published:** 2025-05-21

**Authors:** Meng-Yun Lin, Zhang Zhang, Kathleen Carey, Risha Gidwani, Amresh D Hanchate

**Affiliations:** Department of Social Sciences and Health Policy, Wake Forest University School of Medicine, Winston-Salem, NC 27101, United States; Department of Health Policy and Management, Johns Hopkins Bloomberg School of Public Health, Baltimore, MD 21205, United States; Department of Health Policy and Management, Gillings School of Global Public Health, The University of North Carolina at Chapel Hill, Chapel Hill, NC 27599, United States; Department of Health Law, Policy & Management, Boston University School of Public Health, Boston, MA 02118, United States; Division of Health Care Policy and Research, University of Colorado School of Medicine, Aurora, CO 80045, United States; RAND Corporation, Santa Monica, CA 90401, United States; Department of Social Sciences and Health Policy, Wake Forest University School of Medicine, Winston-Salem, NC 27101, United States

**Keywords:** safety-net providers, value-based payments, health equity, alternative payment models, Medicare

## Abstract

**Introduction:**

Medicare Merit-based Incentive Payment System (MIPS), established by Centers for Medicare & Medicaid Services to transition Medicare reimbursement toward value-based care, has faced criticism for its administrative complexity and potential inequities affecting safety-net providers (SNPs).

**Methods:**

This study analyzed 5-year data (2018-2022) to evaluate the performance and financial outcomes of clinicians consistently participating in MIPS, focusing on disparities between SNPs and non-SNPs.

**Results:**

We found that safety-net specialists were 31% more likely than non–safety-net specialists to consistently receive positive payment adjustments and earned modestly higher average adjustment rates (0.35% points). However, despite this superior performance, safety-net specialists did not achieve greater cumulative financial rewards due to MIPS's percentage-based adjustment structure, which disadvantages clinicians with smaller billing volumes. Our analysis also showed that MIPS financial incentives were generally modest—ranging from $300 to $4000 over 5 years—far below the estimated $12 000 in annual administrative compliance costs per physician reported in prior research.

**Conclusion:**

To address these disparities and inefficiencies, policymakers should consider alternative models such as the American Medical Association's proposed Data-Driven Performance Payment System, which reduces administrative burden by simplifying the reporting process and ensures fairer financial rewards by uncoupling incentive payments from billing volume—thereby improving equity for safety-net clinicians.

## Introduction

The Merit-based Incentive Payment System (MIPS) is a value-based reimbursement system established by the Centers for Medicare & Medicaid Services (CMS) in 2015 to improve care quality by tying Medicare Part B reimbursement to clinician performance. Merit-based Incentive Payment System is the primary track within the Quality Payment Program (QPP), which was created to transition Medicare from fee-for-service to value-based payments. The MIPS program combines several quality reporting programs into a single system, with the goal of encouraging higher-quality care through performance-based payment adjustments.^1,2^ Each year, hundreds of thousands of clinicians across various specialties have opted to remain in MIPS, making it the largest Medicare value-based program in ambulatory care settings.^3^

Implemented in 2017, the MIPS program evaluates clinician performance across 4 areas: quality of care, improvement activities, electronic medical record interoperability, and cost.^4^ Performance in these categories is combined into a composite performance score (CPS) ranging from 0 to 100.^5^ In 2018, CMS introduced the complex patient bonus (up to 10 points added to the CPS) for clinicians who care for a high proportion of medically and socially complex patients.^6^ Each year, CMS sets a performance threshold based on the CPS to determine whether clinicians receive positive, negative, or neutral adjustments to their Medicare Part B payments 2 years later. The performance threshold required to receive a positive adjustment increased from 15 in 2018 to 75 in 2022. Additionally, CMS awards an exceptional performance bonus to clinicians who exceed a higher benchmark for outstanding performance. The program is budget-neutral, meaning that the total positive adjustments (excluding exceptional performance bonuses) are balanced by the total negative adjustments. Starting with a maximum 4% Part B payment adjustment in 2019 (based on 2017 performance), the maximum adjustment gradually increased to 9% in 2022 (based on 2019 performance) and beyond.

Prior evaluations of the MIPS program have focused primarily on annual CPS and factors associated with receiving high scores or positive payment adjustments. Studies have identified clinician characteristics linked to lower scores and negative payment adjustments, including small practice size and high caseloads of minority and socially at-risk patients.^7-12^ Despite the complex patient bonus points, the final CPS score itself is not risk adjusted. Partly as a result, clinicians caring for a higher proportion of socially and medically complex patients, particularly those eligible for both Medicare and Medicaid, tend to receive lower scores. Conversely, clinicians affiliated with healthcare systems or participating in alternative payment models typically achieve higher scores and are more likely to receive positive payment adjustments.^8,9,12,13^

Safety-net providers (SNPs) generally serve more socially disadvantaged individuals and often face resource constraints and staffing challenges.^14,15^ The MIPS program has drawn criticism for both failing to account for patients’ social health factors and creating excessive administrative work.^16-20^ These issues raise concerns that MIPS may unfairly disadvantage SNPs, who treat many minority and low-income patients and likely lack the resources required to report quality measures.^21^ As a result, SNPs may receive lower scores and face financial penalties, which would further strain their limited resources. If MIPS places SNPs at risk for penalties due to the increased complexities and challenges of caring for socially and medically vulnerable populations, it could unintentionally worsen health disparities in underserved communities.

Although prior work has documented cross-sectional MIPS performance disparities among clinicians serving socially and/or medically complex patients, it remains unknown whether these patterns persist as MIPS performance thresholds and payment adjustment rules change. When MIPS introduced a modest risk adjustment through complex patient bonus points in 2018 and doubled its potential financial rewards in 2020, providers with greater technical and staffing capacity—often non-SNPs—are better positioned to improve their performance in response to these higher incentives. To date, no studies have explicitly examined the performance of SNPs under the MIPS program, and evidence on providers’ long-term experiences remains limited. This study fills these gaps by analyzing the longitudinal performance and payment adjustments of SNPs from 2018 to 2022, comparing their outcomes with those of non-SNPs. Specifically, we examined 3 dimensions of clinicians’ long-term MIPS experience: consistent positive payment adjustments across years, average adjustment rates, and total cumulative payment adjustments in dollars over the 5-year period—both overall and by provider type.

## Data and methods

### Data and study sample

Using the Medicare Provider Data Catalog—Doctors and Clinicians,^22^ we identified clinicians consistently participating in MIPS during 2018 to 2022 (to minimize confounding from track switching; see Appendix) and obtained their CPS along with their affiliated practices. We excluded the initial year (2017) from our analysis, as CMS set extremely low performance thresholds (a CPS of 3 to prevent negative payment adjustments) to encourage participation. Some clinicians had multiple CPS scores in a given year due to affiliation with multiple group practices. For these cases, we selected the highest available score, following CMS guidelines.^23^ We linked the MIPS payment adjustment rate and complex patient bonus points for each clinician, as reported in the QPP Experience Report,^24^ using their national provider identifiers (NPIs). We then linked the above data using NPIs and group practice identifiers with the Doctors and Clinicians National Downloadable File^25^ to obtain relevant clinician and practice characteristics, including practice addresses. We geocoded the street addresses of these practices to identify corresponding census block groups. We assigned a 2020 Area Deprivation Index^26^ score to each practice based on its geocoded census block group and a Hospital Referral Region to each practice based on its zip code, using Dartmouth Atlas data for the latter.^27^ We also used NPIs to link information on health system affiliation from the Agency for Healthcare Research and Quality's Compendium of U.S. Health Systems,^28^ clinicians’ average CMS Hierarchical Condition Categories risk scores across their patient panel, and standardized Medicare Part B payment amount from the Medicare Provider Utilization and Payment Data—Physician and Other Supplier Public Use File.^29^

### Identification of SNPs

We identified SNPs using the safety-net status indicator that CMS released in the 2022 QPP Experience Report.^30^ The designation defined SNPs as MIPS-eligible clinicians who rank in the top 20th percentile of all MIPS-eligible clinicians based on their percentage of patients enrolled in both Medicare Part A and Part B as well as full-benefit Medicaid (dual-eligible patients).

### Five-year performance metrics

For each clinician, we evaluated their longitudinal performance across 3 metrics. First, we evaluated whether a clinician continuously received a positive payment adjustment each year from 2018 to 2022 (continuous positive payment adjustment). Second, we calculated the 5-year average of payment adjustment rates (average annual adjustment rate). Lastly, we assessed the total dollar amount of clinicians’ payment adjustments over the 5-year period (cumulative payment adjustment amount). We determined each year's payment adjustment in dollars by multiplying a provider's standardized Medicare Part B payment amount by that year's MIPS adjustment rate. The standardized amount removes geographic variations in payment rates. Since payment adjustments are based on clinicians’ total Medicare Part B payments, which are proportional to their patient volume, we normalized each clinician's cumulative adjustment by dividing it by their annual patient volume.

### Covariates

We constructed covariates reflecting clinicians, practices, and patient characteristics. Clinician characteristics included sex, years of practice (1-5, 6-10, 11+ years), mode of MIPS data submission (individual, group, or alternative payment model entity), and professional type. We defined professional type by categorizing clinicians into 3 groups: primary care physicians (PCPs, including general practice, family practice, internal medicine, and geriatric medicine), advanced practitioners (APs, including nurse practitioners and physician assistants), and specialists. For practice characteristics, we measured practice size (1-5, 6-19, 20-49, 50+ clinicians) and health system affiliation. We also assessed neighborhood socioeconomic disadvantage by grouping practices into quintiles based on their national ranking of the Area Deprivation Index. To account for performance variations due to patient characteristics, we evaluated the clinical complexity of Medicare patients treated by each clinician, using Medicare Provider Utilization and Payment Data.^29^ Clinical complexity was measured by the average CMS Hierarchical Condition Categories risk scores.

### Statistical analysis

Analyses were conducted at the clinician level. We used multivariable regression models to compare longitudinal performance between SNPs and non-SNPs. Logistic regression models assessed continuous positive payment adjustment, while linear regression models evaluated average annual adjustment rate and cumulative adjustment amount. The key independent variable was a binary indicator of safety-net status. These models adjusted for clinician, practice, and patient characteristics that might influence performance, as previously described. Adjustments were also made for the Hospital Referral Region to control geographic variations in practice patterns. We estimated standard errors robust to heteroscedasticity using the Huber-White estimator of variance.^31^

For each outcome, we also conducted subgroup analyses by professional type (PCPs, APs, and specialists) to examine potential heterogeneity in results. All tests were 2-sided with a statistical significance threshold set at *P* < 0.05. We performed analyses using SAS version 9.4 (SAS Institute Inc) and STATA version 18 (StataCorp). This study was deemed exempt by the Institutional Review Board at [REDACTED], with informed consent waived.

### Limitations

This study has several limitations. First, the study sample only included clinicians who continuously participated in the program, which limits generalizability to those who frequently enter and exit MIPS. Second, as this was an observational study, we could not establish a causal relationship between safety-net status and performance in the MIPS program. However, our analysis did adjust for factors known to influence CPS, such as practice size and health system affiliation, as documented in prior research. Third, the absence of patient-level outcome data prevented us from determining whether differences in performance reflect care quality or merely the ability to select and report various MIPS measures effectively.

## Results

### Sample characteristics

The overall sample included 257 548 clinicians who participated in the MIPS program from 2018 to 2022, with 49 128 (19%) identified as SNPs (Tables 1 and Appendix Table A1). Baseline characteristics from 2018 showed that a higher proportion of SNPs participated in MIPS through an alternative payment model entity compared to non-SNPs (33.5% vs 29.0%). Safety-net providers were also less likely to be part of small practices, with 6.9% working in group practices with less than 5 members compared to 9.7% of non-SNPs. Healthcare system affiliation was more common among SNPs (57.2%) than non-SNPs (52.9%). Additionally, a larger proportion of SNPs (21.3%) were primary care physicians compared to 17.5% of non-SNPs. Safety-net providers initially had slightly lower MIPS scores (CPS) than non-SNPs early in the study period but surpassed them after 2019. Yet, the difference remained minimal, never exceeding 4 points. Similarly, SNPs had lower payment adjustment rates in the early years but achieved higher rates later. The difference was small—less than 0.1% points (pp) except in the final year, when it reached 1.6 pp. Despite higher rates, SNPs consistently received lower estimated payment adjustments than non-SNPs. Payment adjustments increased from $918 for SNPs and $1765 for non-SNPs in 2018 to $1908 and $2952, respectively, in 2022. Throughout the study period, SNPs consistently earned higher complex patient bonus points compared to non-SNPs. Safety-net providers earned nearly 4 points in the first year, increasing to around 7 points in later years. In contrast, non-SNPs received 2.5 points in 2018, rising to 4.3 points by 2020 before dropping to 1.5 points by 2022.

**Table 1.** Participation, practice characteristics, and performance of Merit-based Incentive Payment System (MIPS) clinicians by safety-net status, 2018-2022.

	Safety-net providers (SNP)^a^ (*n* = 49 128 clinicians)					Non–safety-net providers (*n* = 208 420 clinicians)				
	2018	2019	2020	2021	2022	2018	2019	2020	2021	2022
Participation type (%)										
Alternative payment model entity	33.5	38.4	40.7	31.4	38.3	29.0	33.1	33.1	23.2	25.4
Group	54.0	50.2	48.1	53.3	50.0	58.0	55.4	56.1	60.3	63.0
Individual	12.6	11.4	11.1	15.3	11.8	13.0	11.5	10.8	16.5	11.6
Practice size (%)										
1-5	6.9	6.9	6.6	6.6	6.4	9.7	8.7	8.2	7.9	7.6
6-19	9.8	11.0	10.1	9.5	9.3	12.5	12.8	12.2	11.4	11.1
20-49	8.4	10.5	10.5	10.1	9.5	11.3	12.0	11.2	10.8	10.3
50+	61.2	64	64.6	66.6	67.8	58.6	60.9	62.6	64.7	66.0
Missing	11.1	7.7	8.2	7.1	7.0	7.9	5.6	5.8	5.1	5.1
Health system affiliation (%)	57.2	−^c^	60.3	64.3	64.5	52.9	−^c^	54.5	55.4	55.3
Provider type^b^ (%)										
Primary care physician	21.3	−^d^	−^d^	−^d^	−^d^	17.5	−^d^	−^d^	−^d^	−^d^
Advanced practitioner	17.1	−^d^	−^d^	−^d^	−^d^	16.1	−^d^	−^d^	−^d^	−^d^
Specialist	61.6	−^d^	−^d^	−^d^	−^d^	66.5	−^d^	−^d^	−^d^	−^d^
Composite performance score (mean)	86.9	86.1	90.2	91.0	86.2	89.2	86.4	89.7	90.7	82.3
Complex patient bonus points (mean)	3.74	3.57	6.64	7.16	6.90	2.55	2.37	4.33	4.22	1.46
Payment adjustment rate (%, mean)	1.16	1.01	1.26	1.59	3.43	1.23	1.07	1.17	1.51	1.83
Payment adjustment (%)										
Negative	2.9	0.1	0.2	0.4	9.6	2.2	0.1	0.3	0.5	12.3
Neutral	1.4	13.6	13.1	14.0	7.4	0.6	13.3	12.6	12.9	9.4
Positive without extra bonus	10.5	0%	3.6	4.8	21.4	8.8	0%	5.5	6.7	37.4
Positive with extra bonus	85.2	86.3	83.1	80.7	61.6	88.5	86.6	81.7	80.0	40.9
Estimated payment adjustment Amount^e^ ($, mean)	918	1077	1033	1336	1908	1765	1665	1607	2211	2952

Source: Authors’ analysis based on data from the CMS Medicare Doctors and Clinicians Datasets and Quality Payment Program Experience Files for performance year 2018 to 2022.

^a^Safety-net providers were defined clinicians who rank in the top 20th percentile of all MIPS-eligible clinicians based on their percentage of dual-eligible patients (based on 2022 QPP Experience Report).

^b^Primary care physicians include general practice, family practice, internal medicine, and geriatric medicine. Advanced practitioners included nurse practitioners and physician assistants.

^c^Not available.

^d^The distributions are the same as at baseline (2018).

^e^The estimated payment adjustment is calculated by multiplying an individual provider's standardized Medicare Part B payment amount by their MIPS payment adjustment rate in a given year. An expanded version of this table that includes SDs, medians, and interquartile ranges for final scores and payment adjustments is available in Appendix Table A1.

### Provider type differences in MIPS scores and payment adjustments

**Figure 1.**
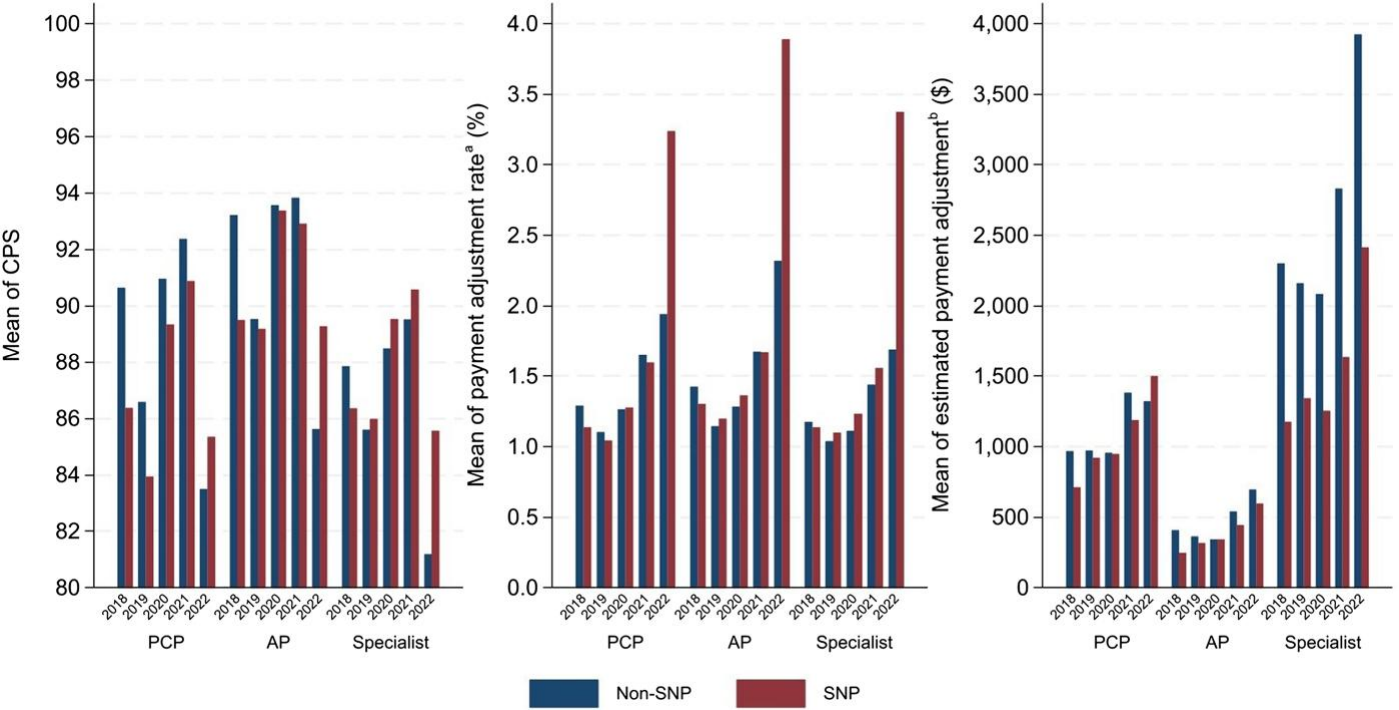
Mean annual performance scores and payment adjustments under the Merit-Based Incentive Payment System (MIPS) program, by safety-net status and provider type, 2018-2022. Source: Authors’ analysis based on data from the CMS Medicare Doctors and Clinicians Datasets and Quality Payment Program Experience Files for performance year 2018 to 2022. ^a^CMS sets a performance threshold each year, which along with clinicians’ annual CPS determines the MIPS payment adjustment rate. ^b^The estimated payment adjustment is calculated by multiplying an individual provider's standardized Medicare Part B payment amount by the MIPS payment adjustment rate in a given year. Primary care physicians (PCPs) include general practice, family practice, internal medicine, and geriatric medicine. Advanced practitioners (APs) include nurse practitioners and physician assistants. CPS, composite performance score; SNP, safety-net provider.

Figure 1 shows trends in CPS, payment adjustment rates, and estimated payment adjustment amounts between 2018 and 2022 for 3 types of providers: PCPs, APs, and specialists. Specialists generally had a lower average CPS than primary care providers, though safety-net specialists scored slightly higher than non–safety-net specialists in most years. Primary care physicians showed the opposite pattern, with non–safety-net PCPs achieving higher scores than safety-net PCPs in most years. For APs, there was no systematic difference between SNPs and non-SNPs. The trend in payment adjustment rates was similar across provider types, rising from 1% to 2.25% in earlier years to over 3% in 2022. Safety-net providers achieved higher payment adjustment rates than non-SNPs in most years, especially among specialists and APs. Estimated payment adjustment amounts, however, varied significantly by provider type: on average, specialists received twice the amounts of PCPs, who in turn received double the amounts of APs. Notably, SNPs consistently received lower payment adjustment amounts than non-SNPs in most years, particularly among specialists. Additionally, the proportion of providers receiving positive adjustments (including those who received extra bonuses) declined over time across all provider types, with a notable drop in 2022 (Appendix Figure A1). However, this decline was more moderate for SNPs compared to non-SNPs.

### Five-year longitudinal performance metrics by safety-net status and provider type

**Figure 2.**
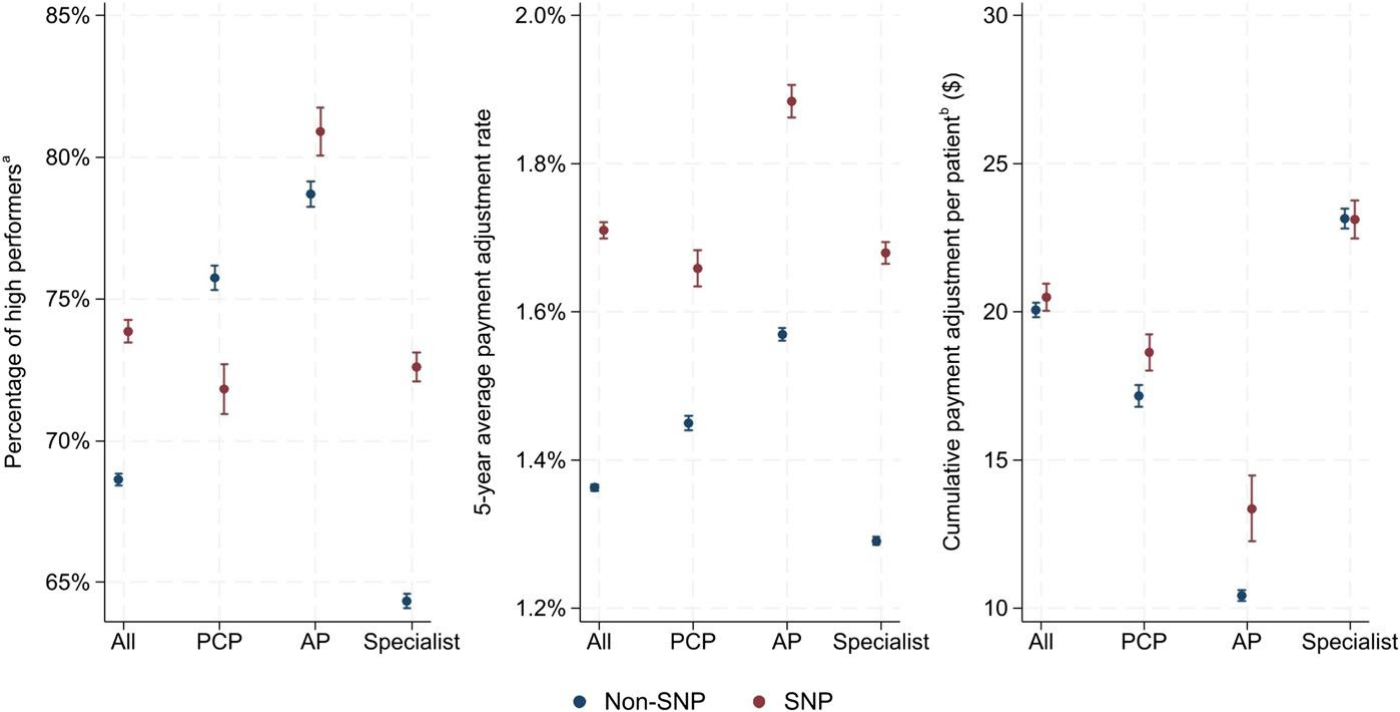
Five-year longitudinal performance of clinicians in the Merit-based Incentive Payment System (MIPS) program by safety-net status and provider type, 2018-2022. Source: Authors’ analysis based on data from the CMS Medicare Doctors and Clinicians Datasets and Quality Payment Program Experience Files for performance year 2018 to 2022. ^a^Clinicians who consistently received positive payment adjustments over the 5-year period. ^b^The 5-year cumulative payment adjustment is normalized by clinicians’ annual patient volume. Each year's estimated payment adjustment is determined by multiplying a provider's standardized Medicare Part B payment by that year's MIPS adjustment rate. Primary care physicians (PCPs) include general practice, family practice, internal medicine, and geriatric medicine. Advanced practitioners (APs) include nurse practitioners and physician assistants. “All” refers to providers of every type (PCPs, APs, and specialists). Dots indicate mean values, with vertical bars representing 95% CIs. SNP, safety-net providers.

Figure 2 compares SNPs and non-SNPs across 3 longitudinal performance metrics by provider type from 2018 to 2022. Overall, SNPs outperformed non-SNPs on 2 metrics, though the magnitude of this difference varied by provider type. Safety-net providers were more likely than non-SNPs to receive positive payment adjustments every year (73.9% vs 68.6%; *P* < 0.001) (Figure 2 and Appendix Table A2). This difference was driven by higher rates among safety-net APs (80.9% vs 78.7%; *P* < 0.001) and specialists (72.6% vs 64.3%; *P* < 0.001), though safety-net PCPs trailed their non–safety-net peers (71.8% vs 75.7%; *P* < 0.001). Safety-net providers also outpaced non-SNPs in mean annual adjustment rates (1.71% vs 1.36%; *P* < 0.001), with a larger difference for APs (1.88% vs 1.57%; *P* < 0.001) and specialists (1.67% vs 1.29%; *P* < 0.001) compared with PCPs (1.66% vs 1.45%; *P* < 0.001) (Figure 2 and Appendix Table A3). These higher rates resulted in slightly larger 5-year per-patient payment adjustments for safety-net PCPs and APs—$18.6 vs $17.2 for PCPs (*P* < 0.001) and $13.4 vs $10.4 for APs (*P* < 0.001). However, the higher payment adjustment rate did not translate to greater dollar payments for safety-net specialists (Figure 2 and Appendix Table A4).

**Figure 3.**
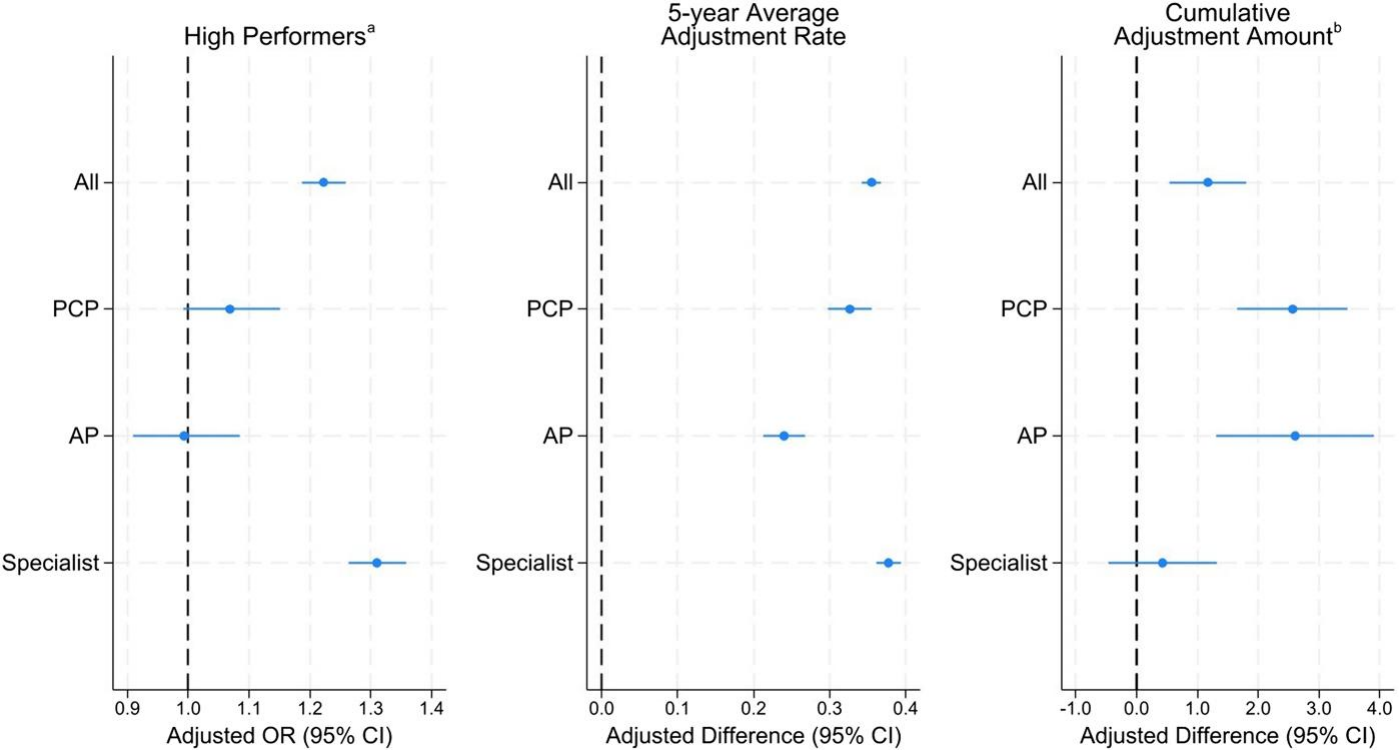
Association of safety-net status with 5-year longitudinal performance under the Merit-based Incentive Payment System (MIPS) program, 2018-2022. Source: Authors’ analysis based on data from the CMS Medicare Doctors and Clinicians Datasets and Quality Payment Program Experience Files for performance year 2018 to 2022. ^a^Consistent receipt of positive payment adjustments over the 5-year period. ^b^Five-year cumulative payment adjustment normalized by clinicians’ annual patient volume. The calculation of annual payment adjustment is described in Figure 2 notes. Adjusted odds ratio (aOR), as estimated by logistic regression, represents the likelihood of safety-net providers consistently receiving positive payment adjustments. Adjusted difference, estimated by linear regression, reflects the differential 5-year average payment adjustment rate and cumulative payment adjustment per patient for safety-net providers relative to non–safety-net providers. All analyses were adjusted for provider sex, years in practice, practice size, health system affiliation, area poverty level, and patient clinical complexity. All, PCP, and AP are described in Figure 2 notes. Statistical significance and estimated 95% CIs are provided in Appendix Tables A2-A4.

Figure 3 shows the associations between safety-net status and 5-year performance metrics, based on regression model results. After adjusting for factors associated with CPS, safety-net status correlated with a slightly higher likelihood of being “high performers” (those continuously receiving positive payment adjustments), higher average annual adjustment rates, and larger cumulative payment adjustments per patient. Safety-net providers were 22.2% more likely (adjusted odds ratio [aOR] 1.222, 95% CI: 1.187, 1.260) than non-SNPs to consistently receive positive payment adjustments under the MIPS program from 2018 to 2022 (Appendix Table A2). This was primarily driven by specialists, with safety-net specialists being 31% more likely (aOR: 1.310, 95% CI: 1.263, 1.359) to be high performers. Safety-net status was linked to a minimally higher average annual adjustment rate across all provider types: 0.33 pp higher for PCPs (95% CI: 0.30, 0.36), 0.24 pp higher for APs (95% CI: 0.21, 0.27), and 0.38 pp higher for specialists (95% CI: 0.36, 0.39) (Appendix Table A3). Furthermore, safety-net PCPs and APs received higher cumulative payments per patient—$2.57 and $2.61 more, respectively—compared to their non–safety-net counterparts (Appendix Table A4).

## Discussion

This study analyzed clinicians’ 5-year performance under Medicare's MIPS program, focusing on how SNPs fared compared to others. Three key themes emerged. First, safety-net specialists were substantially more likely than their non–safety-net peers to receive consistent positive payment adjustments. Yet, this pattern was not observed among other types of SNPs. Second, a misalignment between performance and financial reward was identified for safety-net specialists. Despite their superior performance in consistently securing positive adjustments and achieving higher annual adjustment rates, these advantages did not translate into greater cumulative financial rewards. Third, while estimated payment adjustments were generally modest, they varied significantly across provider types despite only minor differences in CPS and adjustment rates. Notably, specialists received estimated payment adjustments that were 1 to 3 times greater than those awarded to PCPs and APs, despite having slightly lower CPS and adjustment rates.

A growing literature highlights the challenges encountered by providers caring for medically complex and socially vulnerable populations under value-based payment models.^32-37^ Remarkably, we found that safety-net PCPs and APs performed comparably to their non–safety-net counterparts in consistently meeting the thresholds for positive payment adjustments. This finding may be attributable to the complex patient bonus, which awards extra points to providers caring for a high proportion of at-risk patients. Since implementing this bonus in 2018, SNPs have consistently received more bonus points than non-SNPs. These additional points may have helped narrow performance score gaps and allowed SNPs to stay competitive under MIPS, despite serving more complex patient populations. Furthermore, our analysis showed that safety-net specialists were more likely than their non–safety-net peers to receive consistent positive payment adjustments. This suggests that they may have benefited even more from the complex patient bonus than safety-net PCPs and APs. This advantage likely stems from structural differences in measurement design: specialists are primarily evaluated on process-based measures (eg, surgical infection control), whereas PCPs and APs are more often assessed on outcome-based measures (eg, diabetes control). Process measures are typically well-defined, documented in electronic health records, and less dependent on patient behavior. In contrast, outcome measures rely heavily on patient adherence and engagement—factors that are more challenging to achieve in safety-net settings.^38^

Both safety-net PCPs and APs achieved higher annual adjustment rates than their non–safety-net counterparts, resulting in cumulative payment adjustments of $2.6 more per patient over 5 years. Based on their average patient volumes—316 for safety-net PCPs and 185 for safety-net APs—these gains translated into estimated additional payments of $822 and $480, respectively. Safety-net specialists also outperformed their non–safety-net peers with a 0.43 pp higher adjustment rate—the largest difference observed across provider types. However, this edge did not translate into greater financial rewards. This discrepancy underscores a structural limitation: since MIPS adjustments are percentage-based, clinicians with a smaller reimbursement base receive lower absolute dollar increases. Unlike safety-net PCPs and APs, safety-net specialists served significantly smaller patient panels and billed fewer services per patient compared to their non–safety-net counterparts (Appendix Table A5). This led to smaller reimbursement bases for safety-net specialists. As a result, despite higher percentage adjustments, their total payment adjustments remained similar to those of non–safety-net specialists. Our dual metrics—average annual adjustment rates and cumulative per-patient payment adjustments in dollars—reveal how MIPS's percentage-based bonus structure can unfairly disadvantage clinicians with smaller patient panels or lower per-patient billing. Reform proposals—such as the AMA's Data-Driven Performance Payment System (DPPS),^39,40^ which uncouples reward magnitude from billing volume—would help reduce this structural disparity.

The administrative burden of MIPS participation far outweighs its modest financial returns for clinicians. CMS estimates that clinicians collectively spent more than $1.3 billion to comply with MIPS in 2017, followed by an additional $700 million in 2018.^41^ A 2019 study found that practices invested over $12 000 per physician annually to meet MIPS requirements.^18^ In contrast, our study shows that the estimated 5-year cumulative payments for continuously participating clinicians ranged from just $300 to $4000. These modest financial rewards stem from MIPS's budget-neutral design, where performance penalties fund the bonuses without additional funding—limiting potential rewards for high performers. This reveals a significant imbalance: the substantial resources required for reporting and compliance vastly exceed the program's limited financial returns. Our study also uncovers troubling disparities in financial rewards across provider types and safety-net status. Primary care providers (PCPs and APs) receive lower per-patient rewards than specialists, while SNPs earn less than their non–safety-net counterparts. This disparity is concerning because primary care serves as the first point of contact for underserved and at-risk populations, and SNPs treat disproportionately high numbers of patients facing social and economic challenges. Without adequate financial returns to invest in essential services, infrastructure, and care coordination, these providers may struggle to maintain or improve patient outcomes. These challenges, combined with MIPS's high overhead costs, could restrict healthcare access in vulnerable communities, potentially widening existing health disparities. To address these challenges, the AMA's proposed DPPS^39,40^ offers a promising alternative. It aims to reduce administrative burden and compliance costs by streamlining the reporting process (eg, replacing complex checkboxes with simple yes/no attestations), automating quality measurements through claims data, and eliminating costly registry fees. To improve financial returns, DPPS would also separate payment adjustments from total billing amounts, ensuring clinicians are rewarded for quality rather than practice size or revenue.

This study makes 3 new contributions to evaluation of MIPS. First, unlike most prior studies which analyzed single years during the program's initial period,^8,10-13,42^ our analysis of performance and payment adjustments across 5 years (2018-2022) provides a multi-year perspective. An expanded view is necessary because MIPS continuously evolves: the performance threshold for receiving a positive adjustment rose from 15 to 75 between 2018 and 2022 and the maximum payment adjustment rate nearly doubled. Moreover, by following the same cohort, we provide deeper insight into performance trends and impacts on providers. Second, our study identified SNPs and directly compared their outcomes with non-SNPs. Previous studies often defined “high social-risk” clinicians as those in the top quartile of dual-eligible or minority caseload among MIPS participants.^7-11^ This sample-based definition likely misclassified a number of clinicians since many SNPs are excluded from MIPS due to their participation in another QPP track. Instead, we relied on the CMS-defined SNP designation, which identifies the top 20% of all QPP-eligible clinicians based on their percentage of dual-eligible patients. This more reliable identification allows us to provide better evidence on whether MIPS penalizes these providers due to factors such as patient social risk, which are beyond their control. Third, unlike prior studies that focused on annual MIPS scores or the likelihood of receiving positive or negative payment adjustment, we examined cumulative financial outcomes under MIPS. This is a key contribution, as the actual financial impact on clinicians’ sustainability and their ability to invest in quality improvement or infrastructure depends on the total dollar amount received or penalized over time.

Our findings support the calls from professional societies and policy experts to reform MIPS, citing its high administrative complexity, limited financial rewards, poor correlation between performance scores and quality of care, and outsized burden on providers with fewer resources.^37,40,43-46^ These insights come at a pivotal moment as CMS shifts from fee-for-service to quality-linked alternative payment models, with the goal of full implementation by 2030.^47^ CMS continues to update the MIPS program to prepare clinicians for this transition. Starting in 2023, clinicians can participate in the program through MIPS Value Pathways (MVPs)—specialty-focused subsets of measures and activities used to meet program requirements. This new reporting structure aims to reduce the administrative burden on clinicians by aligning measures and activities across the quality, cost, and improvement categories of MIPS that are more relevant to clinicians’ scope of practice. The effectiveness of the MVPs option in differentiating and rewarding exceptional performance remains to be seen. Even if it succeeds, challenges faced by less-resourced providers (such as safety-net and primary care providers) remain unaddressed. These challenges include limited technology vendor support, high costs of ongoing investments, and staffing shortages.^43^ As a result, providers with constrained resources may treat the program as merely a penalty-avoidance exercise rather than an opportunity for quality improvement, undermining the program's core objectives.

## Conclusion

Our findings reveal a misalignment between clinician performance and financial rewards—particularly for safety-net specialists. Though the complex patient bonus has helped level the playing field in performance scores for SNPs, the program's percentage-based payment structure disadvantages clinicians with smaller reimbursement bases. To ensure fairer compensation for clinicians serving complex populations, policymakers should consider reforms that uncouple financial rewards from billing volume, such as the AMA's proposed DPPS or similar alternative. Additionally, CMS needs to evaluate whether MIPS, as currently structured, remains a viable and fair value-based payment model, given its substantial administrative burden and minimal financial returns.

## Supplementary Material

qxaf105_Supplementary_Data
