# Supplementary material for: Merit-Based Incentive Payment System: longitudinal performance and uneven rewards for safety-net providers over 5 years
Source: Health Aff Sch. 2025 May 21;3(6):qxaf105. doi: 10.1093/haschl/qxaf105 (PMC12152720; doi:10.1093/haschl/qxaf105)
Supplement: qxaf105_Supplementary_Data [file qxaf105_supplementary_data.zip › SNP and MIPS_appendix_R1_FINAL-clean.docx]

**Appendix:**

*Rationale for Focusing on Continuous MIPS Participants*

MIPS is one of the two tracks in the Quality Payment Program (QPP), with the other track—the Advanced Alternative Payment Model (AAPM)—carrying higher financial risk for clinicians. Because clinicians can choose between MIPS and AAPM on an annual basis, this study focuses on those who continuously participated in MIPS rather than switching to or remaining in the more financially risky AAPM track. Limiting the sample to consistent MIPS participants allows for clearer attribution of outcomes to ongoing MIPS involvement, reducing the potential confounding effects of track switching or intermittent participation.

The flowchart below shows how many clinicians participated in the MIPS track each year between 2018 and 2022, and the number of clinicians who participated continuously during this period.

Excluded clinicians with missing specialty

(n=673; 0.3%)

2018 MIPS Participating Clinicians

(n=587,498)

2019 MIPS Participating Clinicians

(n=628,622)

2022 MIPS Participating Clinicians

(n=487,135)

2020 MIPS Participating Clinicians

(n=605,698)

2021 MIPS Participating Clinicians

(n=595,988)

Continuous MIPS Participating Clinicians, 2018-2022

(n=258,221)

Study Sample

(n=257,548)

**TABLE A1**

**Participation, Practice Characteristics, and Performance Measures Between Safety-Net and Non-Safety-Net Providers**

|  | **Safety-Net Providers^a^**  **(n=49,128 clinicians)** | | | | | **non-Safety-Net Providers**  **(n=208,420 clinicians)** | | | | |  |
| --- | --- | --- | --- | --- | --- | --- | --- | --- | --- | --- | --- |
|  | **2018** | **2019** | **2020** | **2021** | **2022** | **2018** | **2019** | **2020** | **2021** | **2022** |  |
| **Participation Type** |  |  |  |  |  |  |  |  |  |  |  |
| APM entity | 33.5% | 38.4% | 40.7% | 31.4% | 38.3% | 29.0% | 33.1% | 33.1% | 23.2% | 25.4% |  |
| Group | 54.0% | 50.2% | 48.1% | 53.3% | 50.0% | 58.0% | 55.4% | 56.1% | 60.3% | 63.0% |  |
| Individual | 12.6% | 11.4% | 11.1% | 15.3% | 11.8% | 13.0% | 11.5% | 10.8% | 16.5% | 11.6% |  |
| **Practice Size** |  |  |  |  |  |  |  |  |  |  |  |
| 1-5 | 6.9% | 6.9% | 6.6% | 6.6% | 6.4% | 9.7% | 8.7% | 8.2% | 7.9% | 7.6% |  |
| 6-19 | 9.8% | 11.0% | 10.1% | 9.5% | 9.3% | 12.5% | 12.8% | 12.2% | 11.4% | 11.1% |  |
| 20-49 | 8.4% | 10.5% | 10.5% | 10.1% | 9.5% | 11.3% | 12.0% | 11.2% | 10.8% | 10.3% |  |
| 50+ | 61.2% | 64% | 64.6% | 66.6% | 67.8% | 58.6% | 60.9% | 62.6% | 64.7% | 66.0% |  |
| missing | 11.1% | 7.7% | 8.2% | 7.1% | 7.0% | 7.9% | 5.6% | 5.8% | 5.1% | 5.1% |  |
| **Health System Affiliation** | 57.2% | -^c^ | 60.3% | 64.3% | 64.5% | 52.9% | -^c^ | 54.5 | 55.4 | 55.3 |  |
| **Provider Type**^b^ |  |  |  |  |  |  |  |  |  |  |  |
| Primary care physician | 21.3% | -^d^ | -^d^ | -^d^ | -^d^ | 17.5% | -^d^ | -^d^ | -^d^ | -^d^ |  |
| AP | 17.1% | -^d^ | -^d^ | -^d^ | -^d^ | 16.1% | -^d^ | -^d^ | -^d^ | -^d^ |  |
| Specialist | 61.6% | -^d^ | -^d^ | -^d^ | -^d^ | 66.5% | -^d^ | -^d^ | -^d^ | -^d^ |  |
| **CPS**, mean (SD) | 86.9 (25.3) | 86.1 (18.2) | 90.2 (17.1) | 91.0 (14.2) | 86.2 (20.5) | 89.2 (21.8) | 86.4 (16.2) | 89.7 (16.2 | 90.7 (14.0) | 82.3 (17.2) |  |
| median (IQR) | 100  (87.4-100.0) | 92.5  (83.6-97.1) | 97.9  (89.7-100.0) | 99.0  (89.9-100.0) | 93.6  (82.4-98.1) | 99.4  (91.1-100.0) | 91.5  (84.1-96.0) | 96.4  (89.1-100.0) | 97.4  (89.1-100.0) | 84.9  (76.2-93.0) |  |
| **Complex Patient Bonus Points**, mean (SD) | 3.74 (1.14) | 3.57 (1.16) | 6.64 (2.64) | 7.16 (1.65) | 6.90 (2.91) | 2.55 (0.91) | 2.37 (0.84) | 4.33 (1.84) | 4.22 (1.25) | 1.46 (2.03) |  |
| median (IQR) | 4.00  (3.19-4.51) | 3.63  (3.19-4.34) | 7.00  (6.08-8.34) | 6.92  (6.14-8.22) | 7.50  (5.00-9.16) | 2.58  (2.00-3.00) | 2.45  (1.91-2.90) | 4.56  (3.54-5.50) | 4.14  (3.38-5.02) | 0.00  (0.00-2.79) |  |
| **Payment Adjustment**  **Rate** **(%)**, mean (SD) | 1.16 (1.17) | 1.10 (0.60) | 1.26 (0.73) | 1.59 (0.97) | 3.43 (4.16) | 1.23 (1.03) | 1.07 (0.57) | 1.17 (0.77) | 1.51 (1.01) | 1.83 (3.31) |  |
| median (IQR) | 1.68  (1.09-1.68) | 1.28  (0.68-1.59) | 1.63  (0.66-1.87) | 2.20  (0.88-2.34) | 4.35  (0.66-7.09) | 1.65  (1.26-1.68) | 1.21  (0.71-1.52) | 1.45  (0.59-1.87) | 1.97  (0.77-2.34) | 0.89  (0.10-3.98) |  |
| **Payment Adjustment** |  |  |  |  |  |  |  |  |  |  |  |
| Negative | 2.9% | 0.1% | 0.2% | 0.4% | 9.6% | 2.2% | 0.1% | 0.3% | 0.5% | 12.3% |  |
| Neutral | 1.4% | 13.6% | 13.1% | 14.0% | 7.4% | 0.6% | 13.3% | 12.6% | 12.9% | 9.4% |  |
| Positive without extra bonus | 10.5% | 0% | 3.6% | 4.8% | 21.4% | 8.8% | 0% | 5.5% | 6.7% | 37.4% |  |
| Positive with extra bonus | 85.2% | 86.3% | 83.1% | 80.7% | 61.6% | 88.5% | 86.6% | 81.7% | 80.0% | 40.9% |  |
| **Estimated Payment Adjustment ($)**, mean (SD) | 918 (4647) | 1077 (2694) | 1033 (2941) | 1336 (3997) | 1908 (14440) | 1765 (5820) | 1665 (4870) | 1607 (5485) | 2211 (7156) | 2952 (20386) |  |
| median (IQR) | 512  (149-1380) | 394  (94-1134) | 337  (70-1003) | 430  (85-1238) | 723  (81-2957) | 779  (221-2013) | 565  (134-1593) | 464  (78-1411) | 656  (109-1977) | 398  (15-2138) |  |
| APM: alternative payment model, AP: Advanced practitioner, CPS: Composite Performance Score, SD: standard deviation, IQR: interquartile range. | | | | | | | | | | | |
| ^a^Safety-net providers were defined clinicians who rank in the top 20th percentile of all MIPS-eligible clinicians based on their percentage of dual-eligible patients (based on 2022 QPP Experience Report). ^b^Primary care physicians include general practice, family practice, internal medicine, and geriatric medicine. Advanced practitioners (AP) included nurse practitioners and physician assistants. ^c^Not available. ^d^The distributions are the same as at baseline (2018). | | | | | | | | | | | |

**FIGURE A1**

**Payment Adjustments Under the MIPS Program by Safety-Net Status and Provider Type**

Non-SNPs: Non-safety-net providers, SNPs: Safety-net providers.

Primary care physicians (PCPs) include general practice, family practice, internal medicine, and geriatric medicine. Advanced practitioners (APs) include nurse practitioners and physician assistants.

**TABLE A2**

**Association of Safety-Net Status and Consistent Receipt of a Positive Payment Adjustment (High Performers) Under the MIPS Program During 2018-2022**

|  | SNPs | | non-SNPs | | Unadjusted OR | p-value | Adjusted OR | p-value | 95% CI | |
| --- | --- | --- | --- | --- | --- | --- | --- | --- | --- | --- |
|  | n | mean | n | mean |  |  |  |  |  |  |
| All clinicians | 49,128 | 73.9% | 208,420 | 68.6% | 1.291 | <0.001 | 1.222 | <0.001 | 1.187 | 1.260 |
| PCP^a^ | 10446 | 71.8% | 36,386 | 75.7% | 0.816 | <0.001 | 1.069 | 0.081 | 0.992 | 1.151 |
| AP^b^ | 8,416 | 80.9% | 33,452 | 78.7% | 1.147 | <0.001 | 0.993 | 0.877 | 0.909 | 1.085 |
| Specialist | 30,266 | 72.6% | 138,582 | 64.3% | 1.469 | <0.001 | 1.310 | <0.001 | 1.263 | 1.359 |
| PCP: Primary care physician, AP: Advanced practitioner, SNPs: safety-net providers, non-SNPs: non-safety-net providers, OR: odds ratio, CI: confidence interval. | | | | | | | | | | |
| ^a^Primary care physicians include general practice, family practice, internal medicine, and geriatric medicine. ^b^Advanced practitioners included nurse practitioners and physician assistants. | | | | | | | | | | |

**TABLE A3**

**Association of Safety-Net Status and Average Annual Payment Adjustment Rates Under the MIPS Program During 2018-2022**

|  | SNPs | | non-SNPs | | Unadjusted difference | p-value | Adjusted difference | p-value | 95% CI | |
| --- | --- | --- | --- | --- | --- | --- | --- | --- | --- | --- |
|  | n | mean | n | mean |  |  |  |  |  |  |
| All clinicians | 49,128 | 1.71% | 208,420 | 1.36% | 0.347 | <0.001 | 0.355 | <0.001 | 0.343 | 0.368 |
| PCP^a^ | 10,466 | 1.66% | 36,386 | 1.45% | 0.208 | <0.001 | 0.327 | <0.001 | 0.298 | 0.355 |
| AP^b^ | 8,416 | 1.88% | 33,452 | 1.57% | 0.315 | <0.001 | 0.240 | <0.001 | 0.213 | 0.267 |
| Specialist | 30,266 | 1.67% | 138,582 | 1.29% | 0.389 | <0.001 | 0.377 | <0.001 | 0.361 | 0.394 |
| PCP: Primary care physician, AP: Advanced practitioner, SNPs: safety-net providers, non-SNPs: non-safety-net providers, CI: confidence interval. | | | | | | | | | | |
| ^a^Primary care physicians include general practice, family practice, internal medicine, and geriatric medicine. ^b^Advanced practitioners included nurse practitioners and physician assistants. | | | | | | | | | | |

**TABLE A4**

**Association of Safety-Net Status and Cumulative Payment Adjustments^c^ Under the MIPS Program During 2018-2022**

|  | SNPs | | non-SNPs | | Unadjusted difference | p-value | Adjusted difference | p-value | 95% CI | |
| --- | --- | --- | --- | --- | --- | --- | --- | --- | --- | --- |
|  | n | mean | n | mean |  |  |  |  |  |  |
| All clinicians | 49,128 | 20.5 | 208,420 | 20.1 | 0.43 | 0.100 | 1.17 | <0.001 | 0.54 | 1.81 |
| PCP^a^ | 10,446 | 18.6 | 36,386 | 17.2 | 1.47 | <0.001 | 2.57 | <0.001 | 1.66 | 3.47 |
| AP^b^ | 8,416 | 13.4 | 33,452 | 10.4 | 2.94 | <0.001 | 2.61 | <0.001 | 1.31 | 3.91 |
| Specialist | 30,266 | 23.1 | 138,582 | 23.1 | -0.03 | 0.939 | 0.43 | 0.348 | -0.47 | 1.32 |
| PCP: Primary care physician, AP: Advanced practitioner, SNPs: safety-net providers, non-SNPs: non-safety-net providers, CI: confidence interval. | | | | | | | | | | |
| ^a^Primary care physicians include general practice, family practice, internal medicine, and geriatric medicine. ^b^Advanced practitioners included nurse practitioners and physician assistants. ^c^The five-year cumulative payment adjustment is normalized by clinicians’ annual patient volume. Each year's estimated payment adjustment is determined by multiplying a provider's standardized Medicare Part B payment by that year's MIPS adjustment rate. | | | | | | | | | | |

**TABLE A5**

**Differences in Annual Patient Volume, Service Volume, and Medicare Payment Amounts Between SNPs and non-SNPs**

| **Primary Care Physicians** | 2018 | 2019 | 2020 | 2021 | 2022 |
| --- | --- | --- | --- | --- | --- |
| **# of Medicare Beneficiaries with Part B Services** |  |  |  |  |  |
| Safety-net providers | 351 | 346 | 304 | 305 | 275 |
| Non-safety-net providers | 368 | 371 | 336 | 361 | 321 |
| **# of Part B Services Billed per Patient** |  |  |  |  |  |
| Safety-net providers | 4.4 | 4.5 | 4.4 | 4.4 | 4.3 |
| Non-safety-net providers | 4.5 | 4.5 | 4.3 | 4.3 | 4.2 |
| **Medicare Part B payment Amount ($)** |  |  |  |  |  |
| Safety-net providers | 107,552 | 107,275 | 96,968 | 100,361 | 93,247 |
| Non-safety-net providers | 92,260 | 92,896 | 81,744 | 91,972 | 86,036 |

| **Advanced Practitioners** | 2018 | 2019 | 2020 | 2021 | 2022 |
| --- | --- | --- | --- | --- | --- |
| **# of Medicare Beneficiaries with Part B Services** |  |  |  |  |  |
| Safety-net providers | 203 | 206 | 174 | 175 | 165 |
| Non-safety-net providers | 225 | 235 | 206 | 217 | 208 |
| **# of Part B Services Billed per Patient** |  |  |  |  |  |
| Safety-net providers | 2.5 | 2.7 | 2.7 | 2.8 | 2.7 |
| Non-safety-net providers | 2.5 | 2.6 | 2.5 | 2.6 | 2.4 |
| **Medicare Part B payment Amount ($)** |  |  |  |  |  |
| Safety-net providers | 33,951 | 34,754 | 31,872 | 33,669 | 32,856 |
| Non-safety-net providers | 27,907 | 29,819 | 26,340 | 31,248 | 29,463 |

| **Specialists** | 2018 | 2019 | 2020 | 2021 | 2022 |
| --- | --- | --- | --- | --- | --- |
| **# of Medicare Beneficiaries with Part B Services** |  |  |  |  |  |
| Safety-net providers | 552 | 550 | 452 | 458 | 438 |
| Non-safety-net providers | 733 | 743 | 638 | 663 | 641 |
| **# of Part B Services Billed per Patient** |  |  |  |  |  |
| Safety-net providers | 3.2 | 3.2 | 3.2 | 3.3 | 3.1 |
| Non-safety-net providers | 3.4 | 3.4 | 3.4 | 3.5 | 3.2 |
| **Medicare Part B payment Amount ($)** |  |  |  |  |  |
| Safety-net providers | 125,261 | 125,841 | 107,062 | 112,326 | 104,994 |
| Non-safety-net providers | 149,918 | 153,694 | 133,201 | 143,908 | 136,227 |
